# MnDPDP: Contrast Agent for Imaging and Protection of Viable Tissue

**DOI:** 10.1155/2020/3262835

**Published:** 2020-09-04

**Authors:** Per Jynge, Arne M. Skjold, Ursula Falkmer, Rolf G. G. Andersson, John G. Seland, Morten Bruvold, Viggo Blomlie, Willy Eidsaunet, Jan O. G. Karlsson

**Affiliations:** ^1^Department of Radiology, Innlandet Trust Hospital, Gjøvik Hospital, Gjøvik, Norway; ^2^Department of Radiology, Helse Fonna, Haugesund Hospital, Haugesund, Norway; ^3^Department of Oncology, University Hospital, Aalborg, Denmark; ^4^Division of Drug Research/Pharmacology, Linköping University, Linköping, Sweden; ^5^Department of Chemistry, University of Bergen, Bergen, Norway; ^6^GE Healthcare, Oslo, Norway; ^7^IC Targets AS, Oslo, Norway

## Abstract

The semistable chelate manganese (Mn) dipyridoxyl diphosphate (MnDPDP, mangafodipir), previously used as an intravenous (i.v.) contrast agent (Teslascan™, GE Healthcare) for Mn-ion-enhanced MRI (MEMRI), should be reappraised for clinical use but now as a diagnostic drug with cytoprotective properties. Approved for imaging of the liver and pancreas, MnDPDP enhances contrast also in other targets such as the heart, kidney, glandular tissue, and potentially retina and brain. Transmetallation releases paramagnetic Mn^2+^ for cellular uptake in competition with calcium (Ca^2+^), and intracellular (IC) macromolecular Mn^2+^ adducts lower myocardial *T*_1_ to midway between native values and values obtained with gadolinium (Gd^3+^). What is essential is that *T*_1_ mapping and, to a lesser degree, *T*_1_ weighted imaging enable quantification of viability at a cellular or even molecular level. IC Mn^2+^ retention for hours provides delayed imaging as another advantage. Examples in humans include quantitative imaging of cardiomyocyte remodeling and of Ca^2+^ channel activity, capabilities beyond the scope of Gd^3+^ based or native MRI. In addition, MnDPDP and the metabolite Mn dipyridoxyl diethyl-diamine (MnPLED) act as catalytic antioxidants enabling prevention and treatment of oxidative stress caused by tissue injury and inflammation. Tested applications in humans include protection of normal cells during chemotherapy of cancer and, potentially, of ischemic tissues during reperfusion. Theragnostic use combining therapy with delayed imaging remains to be explored. This review updates MnDPDP and its clinical potential with emphasis on the working mode of an exquisite chelate in the diagnosis of heart disease and in the treatment of oxidative stress.

## 1. Background

MRI is an imaging modality which in its native form produces important diagnostic information with purely instrument-based techniques [1]. Diagnostic routine on the other hand commonly relies upon the use of intravenous (i.v.), extracellular (EC) contrast agents containing gadolinium (Gd). At present, new contrast agent free (native) techniques are advancing into clinical practice whereas a strong standing of Gd agents seems reaffirmed after linear and semistable chelates were discarded and by paying attention to kidney function [2].

Still, there is a demand for new contrast enhancing techniques with properties beyond the scope of both native and Gd based MRI. Especially, there is a high need for agents that enable imaging and quantification of tissue viability at a cellular or close to molecular level. In addition to ensuring efficacy and safety, preferred new agents should be able to improve upon the treatment of patients undergoing diagnostic imaging. In retrospect, such an agent, manganese (Mn) dipyridoxyl diphosphate (MnDPDP), has already been available but vanished before its potential was recognized by the imaging community.

**Figure 1:**
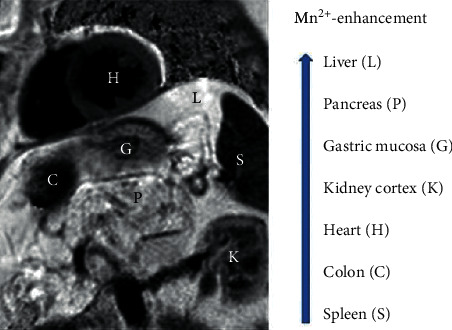
MnDPDP: *T*_1_ weighted image (*T*_1_WI) of heart and abdominal organs. Signal intensity (SI) in Mn^2+^-enhanced tissue increases from spleen to liver. Imaging 60 min after i.v. infusion of MnDPDP 5 *μ*mol/kg in a patient with a recent acute myocardial infarction (AMI) located to left ventricular (LV) septum (Skjold A, unpublished data).

Paramagnetic Mn^2+^ was the first metal ion studied for contrast enhancement in MRI [3], but fear of cardiotoxicity and rapid progress of Gd agents restrained the development of Mn-ion-enhanced MRI (MEMRI) [4, 5]. As a consequence, MnDPDP (Teslascan™, GE Healthcare, Oslo, Norway) became the only i.v. Mn agent for human use (Figure 1) approved for imaging of liver and pancreas [6, 7]. After a decade low product earning led to cessation of marketing (USA 2003) or direct market withdrawal (Europe 2011). At that time intracellular (IC) Mn^2+^ was recognized as an excellent biomarker of cellular events in various tissues and organs including heart and brain, but mainly in animals [8–15] and only partly in humans [16–19]. In parallel, human studies of MnDPDP and its key metabolite MnPLED (Mn dipyridoxyl diethyl-diamine) as small molecular catalytic antioxidants controlling reactive oxygen and nitrogen species (ROS, RNS) were in an early phase [20–25].

The aims of the present review are twofold: to focus on a multifunctional chelate with highly differing functions and mechanisms (basic properties) and with early examples from human use to indicate its future possibilities in MEMRI and therapy (application in humans).

## 2. Basic Properties

The behavior of MnDPDP as chelated prodrug in medical biology represents a blend of disciplines, ranging from physics and chemistry to pharmacokinetics and physiology in health and disease. From traversing these fields, come the basics of MRI and of antioxidant treatment. In spite of an inherent complexity, interactions between multiple factors seem mostly fortuitous.

### 2.1. Physics and Chemistry: In Vitro and In Vivo Factors

**Figure 2:**
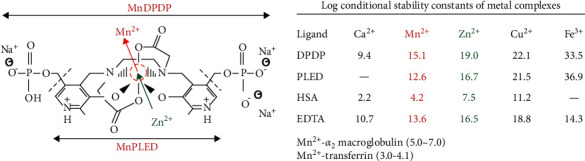
MnDPDP: structure, transmetallation, and stability. In MnDPDP 3 anionic sites are balanced by 3 sodium ions. MW: MnDPDP ∼680 Da, MnPLED ∼520 Da. Transmetallation mainly by zinc (Zn^2+^) releases Mn^2+^. The enclosed table presents log conditional stability constants for metal complexes with DPDP, PLED, HSA (human serum albumin), and EDTA (ethylene-diamine tetra-acetic acid). Log values for Mn^2+^ binding to main transport proteins in plasma are also included. Material derived from [26–29].

MnDPDP (mangafodipir) is a hexadentate and linear chelate in which a dimer of vitamin B_6_ (pyridoxal phosphate) forms a metal binding pocket (Figure 2). In this site Mn^2+^ shares 5 unpaired electrons with 4 oxygen and 2 nitrogen atoms of DPDP (fodipir) and may undergo reversible one-electron oxidation-reduction [26–28]. The 5 unpaired electrons of Mn^2+^ yield a strong magnetic moment (5.9 BM (Bohr Magnetom)) while Mn^3+^ with 4 is weaker (4.9 BM) and gadolinium (Gd^3+^) with 7 is considerably stronger (7.6 BM). Electron spin resonance (ESR) time is longer and more optimal with Mn^2+^ and Gd^3+^ (10^−8^–10^−9^ s) than with Mn^3+^ (10^−10^–10^−12^ s). The *in vitro* molar longitudinal relaxivity (*r*_1_) is 4 times higher with MnCl_2_ than MnDPDP.

A prerequisite for diverse functions of MnDPDP and MnPLED is a chelator being able to release and bind biologically active metal ions in a highly hierarchic manner (Figure 2). Accordingly, with DPDP and PLED the log conditional stability constants [27], a main index of metal-chelator affinities, for Mn^2+^ are well above those of calcium (Ca^2+^) and magnesium (Mg^2+^) but also well below those of zinc (Zn^2+^) and of copper (Cu^2+^) and far below those of iron (Fe^3+^). Accordingly, in tissue compartments MnDPDP and MnPLED undergo successive transmetallation steps depending mainly on chelator-metal affinities (log values) and only partly on concentrations of Mn^2+^ and competing cations [26–29]. Of prime importance is that Mn^2+^ displaces Ca^2+^ from binding to physiological ion channels in the cell membrane and to IC storage and release sites.

Zn^2+^, with log value of 19.0 (16.7) in binding to DPDP (PLED) and relative abundance in plasma and interstitium, is a powerful transmetallator of Mn^2+^ with 15.1 (12.6) and, in retrospect, also of Gd^3+^ in gadodiamide with reported 14.9 [30]. With far higher log values, traces of Fe^2+^ may outstrip any other endogenous cation from binding to DPDP and PLED. Accordingly, i.v. administration of MnDPDP in humans caused a transient fall not only in plasma Zn^2+^ [29] but also in serum Fe^2+^ [6], with bottom reached at 2 hours and returning to baseline at 24 hours. Furthermore, a comparison with clinical chelators reveals that the *in vitro* log values of FeDPDP (33.5) and FePLED (36.9) [27] are as high as or even higher than those reported for, respectively, deferoxamine (31) and deferitazole (33.4) [31].

Like other metal ions, endogenous Mn^2+^ appears bound, mainly to large molecules in plasma and cytosol and in organelles where Mn^2+^ attains catalytic functions [32–34]. A model role is shown in mitochondrial superoxide dismutase (SOD) containing Mn^2+^-Mn^3+^ as redox pair in its catalytic site (MnSOD). Another consequence of macromolecular binding is an increase in the rotational correlation time between Mn^2+^ and protons in water, thereby greatly enhancing *r*_1_ of potential Mn^2+^adducts [26].

### 2.2. Biotransformation in Human Volunteers

**Figure 3:**
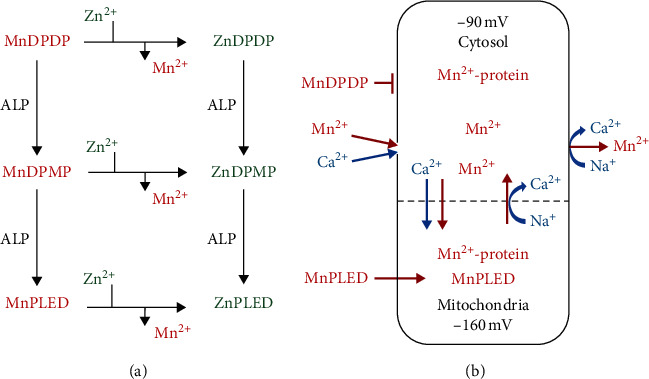
MnDPDP: metabolism and Mn^2+^ uptake and retention in excitable cells. (a) MnDPDP is metabolized in plasma, microcirculation, and interstitium by transmetallation, mainly with Zn^2+^, and by the action of alkaline phosphatase (ALP) before delivering Mn^2+^ and MnPLED for cellular uptake. (b) Mn^2+^ follows Ca^2+^ and electrochemical gradients into and out of cardiomyocytes. Lipid soluble MnPLED is able to enter cells as intact agent. IC Mn^2+^ retention for hours is caused by macromolecular binding, especially in protein-dense mitochondria, and by a slow efflux via bidirectional Na^+^/Ca^2+^ exchangers (NCXs). Material derived from [29, 35–37].

According to a thorough review by Toft et al. [29], i.v. administered MnDPDP distributes and releases active metabolites in plasma and interstitium (Figure 3(a)). In one pathway, µmolar Zn^2+^ transmetallates 75–80% of Mn^2+^ in a clinical dose of MnDPDP (5–10 *μ*mol/kg) for stepwise uptake in target cells. After bolus injection of 5 and 10 *μ*mol/kg, about 20% of Mn^2+^ is released within 2 min by µmolar Zn^2+^ present in plasma. Thereafter about 50% is released in a delayed manner by gradually available Zn^2+^ and possibly by millimolar Ca^2+^ and Mg^2+^ within the interstitial space.

In another pathway, alkaline phosphatase (ALP) in plasma and microcirculation [29] converts water soluble MnDPDP (ZnDPDP) via monophosphate MnDPMP (ZnDPMP) to lipid soluble MnPLED (ZnPLED). Dephosphorylation enables MnPLED to diffuse across cell membranes and even enter organelles like mitochondria. The 20–25% end product MnPLED disappears from plasma over 60–90 min, whereas ZnPLED remains detectable up to 8 hours. Elimination from the body differs between Mn^2+^ and its ligands [29]. The liver acts as a Mn^2+^ sink with rapid turnover from plasma and hepatobiliary excretion, while the chelating moieties undergo renal elimination. The administered Mn is recovered within 1-2 hours (urine 25%), few days (feces 50–60%), and weeks (feces).

### 2.3. Cell Mn^2+^ Uptake and Competition with Ca^2+^

Mn^2+^ is a most potent stalker of Ca^2+^, conductor of both cell function and energy metabolism. Hence a graded Mn^2+^ uptake and retention in cardiomyocytes and other excitable cells (Figure 3(b)) mirror the activity of Ca^2+^ transporters and IC ligands to which Mn^2+^ has a higher affinity [8, 9, 12, 35–37]. Mn^2+^ entry into target cells like cardiomyocytes occurs predominantly via voltage dependent L-type Ca^2+^ channels that open briefly during depolarization [35]. Transient receptor potential (TRP) operated Ca^2+^ (and Na^+^) channels [37] and bidirectional Na^+^/Ca^2+^ exchangers (NCXs) [36] may also mediate Mn^2+^ influx or retention, probably more in injured than normal cardiomyocytes or in myofibroblasts during repair. Mitochondrial Mn^2+^ entry is via a Ca^2+^ uniport and exit from mitochondria and cytosol occurs via NCXs. Neuronal Mn^2+^ uptake occurs via N-type Ca^2+^ channels but requires prior transport over the blood-brain-barrier (BBB) and diffusion via cerebrospinal fluid [14–16]. Divalent metal ion transporters [38] are active in longer term cell exchange of Mn^2+^.

The use of Mn^2+^ as a Ca^2+^ analog to study normal physiology and contrast enhancement in the animal heart and brain have been highlighted in reviews not dealt with here [8, 9, 12–15]. However, repeated notions of MnDPDP being a cardiotoxic agent still deserve comment [4, 8]. Thus the high affinity to Ca^2+^ channels may in theory depress cardiovascular function during high dose and rapid i.v. administration of Mn^2+^-releasing agents. This was exemplified by Wolf and Baum with MnCl_2_ in anesthetized animals in the early days of MRI [4].

Later studies by Jynge et al. in isolated buffer-perfused small animal hearts [8, 39, 40] confirmed that myocardial Mn content and longitudinal relaxation rate (*R*_1_) correlated positively with perfusate [Mn^2+^] and negatively with left ventricular (LV) developed pressure (LVDP); i.e., high dose Mn^2+^ acts as cardiodepressor. Importantly, with perfusate [Mn^2+^] below 30 *μ*M, LVDP was not affected but still tissue Mn content rose 5 times and *R*_1_ 2.5 times; i.e., there is a wide margin for diagnostic efficacy without cardiodepression. Since interstitial [Mn^2+^] after clinical doses of MnDPDP in humans is probably less than 1–5 *μ*M [41] and, in nonmedicated conscious dogs, high plasma [Mn^2+^] may activate adrenal release of catecholamines [8], negative inotropy and hypotension will hardly occur in humans. This is also confirmed by broad clinical experience [6, 7, 17–19, 22–25].

### 2.4. Safety and Brain Accumulation

MnDPDP has an about 10 times higher safety margin than MnCl_2_ reflecting a more gradual release of Mn^2+^ [8]. In offspring of rats both agents produced skeletal defects related to Mn only [42]. Mn^2+^-releasing agents are thus contraindicated in early pregnancy and preferably in patients with pheochromocytoma. In humans, mild transient side effects mediated by nitric oxide (NO) [20, 43] like flushing, occasional headache, and mild diarrhoea are observed during high dose infusion or rapid injection of MnDPDP [6].

In the adult human body, the Mn content, 10–20 mg (182–364 *μ*mol) [32, 33], is in the order of an imaging dose. Still transient accumulation in most tissues seems to be well tolerated. An important exception is the brain where a transient and limited Mn^2+^ uptake may become a safe tool in functional MRI while a persistent Mn elevation in basal ganglia may induce oxidative injuries. Also Parkinson-like symptoms are feared outcomes from long term exposure to Mn metal whether being environmental, following total parenteral nutrition, or being caused by liver failure [33, 44, 45]. Importantly, with MnDPDP, single doses up to 25 *μ*mol/kg were applied in phase II trials without reported signs of Parkinsonism [6], and based on the success with MEMRI for study of brain physiology in animals [14, 15] Reich and Koretsky are exploring the possibility of using MnDPDP to image neuronal activity and neural tracts in patients with multiple sclerosis [46]. However, Sudarshana et al. recently reported [47] that i.v. infusion of a standard imaging dose (5 *μ*mol/kg) of MnDPDP in healthy human volunteers raised signal intensity (SI) in exocrine glands in the head and neck, in the choroid plexus, and in the anterior pituitary gland but not beyond the intact BBB.

### 2.5. MEMRI and Contrast Enhancement

MR properties of IC Mn^2+^, as the agent that ultimately shortens longitudinal relaxation time (*T*_1_ = 1/*R*_1_) but to a lesser degree transversal relaxation time (*T*_2_ = 1/*R*_2_) of excited protons, have been studied mostly with use of MnCl_2_ as Mn^2+^-delivering agent. Main mechanisms influencing efficacy of Mn^2+^ enhancement in a highly excitable tissue like the LV myocardium have been comprehensively analyzed by Seland et al., Hu et al., and Bruvold [48–50]. Using relaxography to examine small animal hearts, mostly additive factors related to *T*_1_ behavior, *R*_1_-Mn relationships, macromolecules, and field dependence were studied.

#### 2.5.1. Monoexponential *T*_1_ Relaxation

In the rat heart, a high transmembrane water exchange rate (∼10 s^−1^) caused tissue *T*_1_ relaxation, representing the sum of IC and EC water protons, to become monoexponential. Only after an extreme Mn^2+^ overload was a second, probably mitochondrial, *T*_1_ peak disclosed.

#### 2.5.2. Correlation between *R*_1_ and Mn Content

A linear correlation was found between tissue *R*_1_ and Mn content up to about 10 times normal, i.e., from about 45 to about 500 *μ*mol/kg dry wt. This makes *R*_1_ a reliable parameter of Mn^2+^ uptake and cell function whereas MEMRI of mitochondria, otherwise an exciting target, becomes less likely without supplementary MR techniques [48, 50]. As expected, the about one order of magnitude higher *R*_1_ of bound *vs*. free Mn^2+^ makes MEMRI possible with a low µmolar dose of a Mn^2+^-releasing agent.

#### 2.5.3. Magnetic Dispersion and Resolution: Low vs. High Field Imaging

A limitation is that magnetic dispersion above 0.2–0.5 Tesla (T) [24] reduced tissue *r*_1_ (s^−1^·mM^−1^) from 40–50 at 0.5 T, to 30–35 at 2.35 T, and to 20–25 at 7 T [48, 50]. Conversely, compensating for a reduction in *r*_1_ of Mn^2+^ adducts at higher fields, the signal to noise ratio (SNR) in *T*_1_ weighted images (*T*_1_WI) increases by at least one order of magnitude. Furthermore, the scale for measuring tissue *T*_1_ expanded by about 30% (native gain) and 40% (Mn^2+^-enhanced gain) when raising the field strength from 0.5 T to 7.0 T [26, 50].

Taken together, MEMRI with IC Mn^2+^ adducts can be applied for both low (0.5–1.5 T) and high field (3.0–7.0 T) imaging. In the heart, a further advantage is that MEMRI may comply with and improve upon recent and impressive achievements in native *T*_1_-based methods [51, 52].

#### 2.5.4. MEMRI vs. Gd-Based MRI

**Figure 4:**
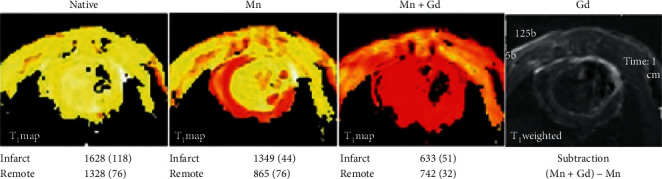
Dual contrast imaging with IC Mn and EC Gd in infarcted rat myocardium. Rats with permanently ligated left coronary artery underwent single session cardiac MRI at 7.0 T. The figure displays *T*_1_ maps of LV myocardium: Native; Mn (MnCl_2_ infusion (25 *μ*mol/kg)); and Mn + Gd (gadodiamide injection 150 *μ*mol/kg)). At the end of the experiment, Gd was obtained by late (10 min) Gd-enhancement and subtraction technique (*T*_1_WI). *T*_1_ values in msec (mean (SD)) are included. IC Mn adducts lower *T*_1_ mainly, but not exclusively, in viable cardiomyocytes whereas EC located gadodiamide lowers *T*_1_ and raises SI mainly inside infarcted tissue (Bruvold M, Seland JG, Jynge P, unpublished data 2006).

The efficacy of MEMRI is, as expected, also highly influenced by physiologic and pharmacokinetic factors which differ from Gd based MRI. In theory, IC Mn^2+^ uptake requires an active metabolism and function and requires that healthy cells retain Mn^2+^ by strong IC binding and slow efflux. Contrary to this, EC Gd agents accumulate briefly within the interstitial, including disrupted IC, water phase. Consequently, when measuring myocardial infarct size (IS) in rats with permanent coronary artery ligation (Figure 4), IC Mn^2+^ adducts lower *T*_1_ mainly in viable cardiomyocytes while Gd-complexes do so in dead or severely injured tissue (Bruvold M, Seland JG, Jynge P, unpublished material).

### 2.6. Tissue Protection in Oxidative Stress

Following a side track from contrast agent research into the field of “oxidative,” i.e., combined oxidative-nitrosative, stress and antioxidants [53–55], Asplund et al. discovered that MnDPDP and MnPLED dilated arteries [20] by mimicking MnSOD, with the proposed mechanism that suppression of superoxide preserved endothelial derived NO for activation of adenylate cyclase and cyclic GMP thereby relaxing vascular smooth muscle cells [43]. Thereafter, electron paramagnetic resonance (EPR) spectroscopy with MnDPDP and MnPLED [21] added to an *in vitro* superoxide-generating (xanthine oxidase) reaction proved that they mimic MnSOD [34] with a half maximal response concentration (EC_50_) of 5–10 *μ*M, a highly relevant plasma level in humans [29]. MnSOD inactivates superoxide (O_2_^−^) leaking from the electron chain by instant dismutation to hydrogen peroxide (H_2_O_2_) and O_2_. Zn-ligands were without SOD activity.

**Figure 5:**
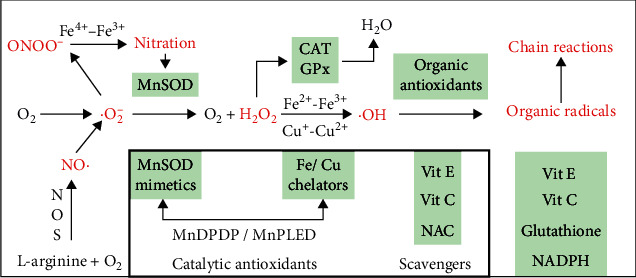
ROS-RNS with intrinsic cell defence (outer box) and exogenous antioxidants (inner box). The diagram presents free radicals with unpaired electrons (.marked) and other oxidizing byproducts of respiration. Secondary pathways activated by ROS-RNS are not included. Observe the dependence of NO· upon MnSOD and H_2_O_2_ upon CAT and GPx or upon binding of prooxidant Cu^+^ and Fe^2+^. Suboptimal control of *·*O_2_^−^ and Fe^2+^ or Cu^+^ may release highly toxic ONOO^−^ and ·OH, radicals which initiate protein nitration and secondary chain reactions attacking most cell constituents. The strategic position of MnDPDP/MnPLED as direct (MnSOD mimetic) and indirect (Fe^2+^/Cu^+^ chelation) catalytic antioxidants is indicated. Material derived from [34, 43, 53–59]. *·*O_2_^−^, superoxide; H_2_O_2_, hydrogen peroxide;·OH, hydroxyl radical; NO·, nitric oxide; ONOO^−^, peroxynitrite; NOS, nitric oxide synthase; MnSOD, mitochondrial SOD; CAT, catalase; GPx, glutathion peroxidase; NAC, N-acetyl-cysteine; scavengers, antioxidants consumed by ROS-RNS and chain reactants in a one-to-one manner.

Experimental data indicate that both EC MnDPDP and IC MnPLED can be characterized as small molecular enzyme mimetics endowed with catalytic antioxidant properties (Figure 5). In acute or subacute conditions of oxidative stress and inflammation, they seemingly act in either of two ways: by supplementing SOD activity in plasma and IC and by binding prooxidant metals like Cu^+^ and Fe^2+^ which leak from IC sites [34, 56–59]. MnDPDP and MnPLED may thereby improve the balance between salient (low-level) and damaging (high-level) ROS-RNS: by preserving NO and hydrogen peroxide for cell signaling [53, 54, 59] and by inhibiting release of superoxide, hydroxyl (OH), and peroxynitrite (ONOO^−^) [43, 55, 56, 59]. Other secondary mechanisms may include stabilization of lysosomes and mitochondria [60, 61]. Altogether, these properties make MnDPDP a promising drug delaying tissue injury and inhibiting inflammatory responses. A further implication of strong chelator binding of Fe^2+^, besides inhibiting oxidative stress in severe inflammation, is an apparent potential to slow replication of rapidly dividing malignant cells [58, 62] and microorganisms [63].

**Figure 6:**
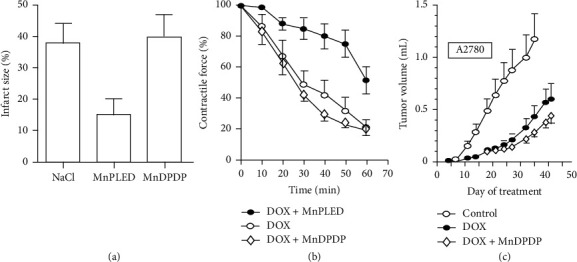
Therapy with MnDPDP: preclinical examples. (a) Reperfusion after AMI [10]. In anesthetized pigs MnPLED, but not MnDPDP and NaCl (placebo), infused i.v. prior to and during reperfusion reduced infarct size at the end of the experiments. Reversible ligation of left coronary artery ligation with 30 min ischemia and 120 min reperfusion (reprinted with permission from Acta Radiol). (b) Cardioprotection during chemotherapy with doxorubicin (DOX) [58]. MnPLED but not MnDPDP improved inotropy during in vitro exposure to toxic doses of DOX. Water bath model with paced left atrial preparations excised from mice after pretreatment with MnDPDP (10 *μ*M) or MnPLED (10 *μ*M). Groups: DOX alone; DOX + MnDPDP; DOX + MnPLED (reprinted with permission from Transl Oncol). (c) Antitumoral efficacy of doxorubicin (DOX) [58]. Human ovarian tumor (A2780) bearing nude mice were treated with repeated cycles of DOX and prior infusion of MnDPDP. At the end of the study, DOX alone (control) significantly reduced tumor volumes by about 50%. There was a tendency that MnDPDP increased the antitumoral effect of DOX (reprinted with permission from Transl Oncol).

In preclinical studies, MnDPDP and/or MnPLED provided significant cytoprotection in chemotherapy of cancer [62, 64, 65], liver failure during paracetamol poisoning [66], the heart and liver during reoxygenation/reperfusion after hypoxia/ischemia [10, 21, 67], and graft protection in transplantation of liver [68]. In AMI in pigs (Figure 6(a)), MnPLED, but not MnDPDP, ameliorated ROS-RNS inflicted reperfusion injury, thereby reducing infarct size by 55%, whereas both agents prevented arrhythmias [10]. These findings imply that MnPLED accessed mitochondrial sites critical for cell survival [61] and that MnDPDP may have acted at the cell membrane level.

Radiation and anticancer drugs produce ROS-RNS [64, 65, 69, 70], and preclinical studies have shown that MnDPDP and/or MnPLED may protect nerve cells, leukocytes, lymphocytes, and cardiomyocytes against toxicity of anticancer drugs (anthracyclines, taxanes, and platinum agents) apparently without loss of anticancer activity [24, 58, 59, 64, 65]. In mice, MnPLED preserved myocardial function (Figure 6(b)) during ex vivo exposure to doxorubicin, and MnDPDP tended to enhance in vivo tumor reduction (Figure 6(c)) by the same agent [58].

## 3. MEMRI in Humans

As amply documented in animals and partly confirmed in humans, MEMRI enhances tissue contrast by Mn^2+^ uptake and retention in excitable cells in liver, pancreas, kidney cortex and medulla, myocardium, endocrine and exocrine glands, and potentially retina and brain [4, 8–19, 39, 40]. With MnDPDP, preclinical studies were frequent prior to or just after the millenium shift, and readers are referred to comprehensive reviews from that time [8, 9, 11, 13, 16, 39, 40]. In patients, MnDPDP, i.e., Teslascan™, has been successfully applied for diagnostic imaging of diseases in liver and pancreas where it demonstrated efficacy in detecting tumor lesions including metastatic disease [7]. Off-label use has mainly included cardiac imaging in human volunteers [16–18, 71] and in patients with ischemic cardiomyopathy [19, 72–74]. These early examples in MEMRI are detailed as follows.

### 3.1. *T*_1_ (*R*_1_) Mapping of Myocardium with MnDPDP

**Figure 7:**
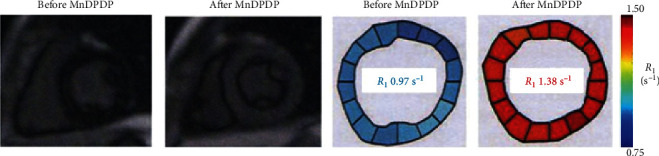
MnDPDP: cardiac MEMRI in a healthy human volunteer [17]. Short axis *T*_1_WI and *R*_1_ maps before (native) and 60 min after i.v. infusion of MnDPDP 5 *μ*mol/kg are presented. Imaging at 1.5 T. Mean *T*_1_ values of 16 sectors were before MnDPDP 1030 ms and after MnDPDP 725 ms (reproduced with permission from J Magn Reson Imaging).

In studies by Skjold et al., *T*_1_ mapping and *T*_1_ weighted imaging (*T*_1_WI) were applied to short axis slices of LV myocardium (Figure 7) before and after *i.v.* infusion of MnDPDP (range 5–15 *μ*mol/kg) [17–19, 74]. *T*_1_ was measured at 1.5 T (Siemens Magnetom Symphony) by use of an inversion recovery (IR) technique [75, 76] with an IR turbo fast low-angle shot (FLASH) sequence and inversion times (TI) ranging from 90 to 5000 ms. Mean values from multiple regions of interest (ROIs) were processed into one mean *T*_1_ (*R*_1_) value representing each of 16–24 transmural LV sectors within a myocardial 8 mm thick slice. In healthy volunteers (*N* = 25) mean values of native *T*_1_ in LV cavitary blood (∼1540 ms) were similar to and in LV myocardium (∼1020 ms) 7% higher than those reported in a more representative reference population (*N* = 342) for native *T*_1_ mapping at 1.5 T [77].

### 3.2. Dose-Response and Mn^2+^ Retention

**Figure 8:**
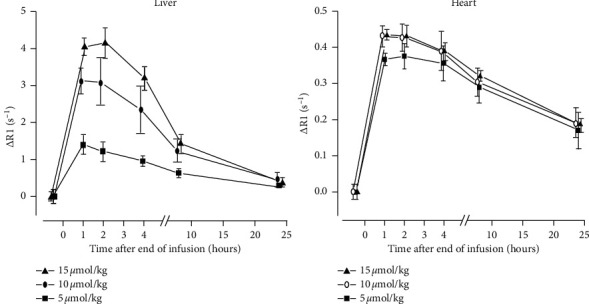
MnDPDP: dose-response and Mn^2+^ uptake/retention [17]. *R*_1_ was measured at 1.5 T in liver and LV myocardium before and after MnDPDP (5, 10, or 15 *μ*mol/kg) administered outside magnet. Δ*R*_1_ values are displayed (reproduced with permission from J Magn Reson Imaging).

In human volunteers, as measured by Wang et al. [16], there is an ascending signal intensity (SI) in *T*_1_WI from minimal in spleen to maximal in kidney cortex, pancreas, and liver following imaging doses (5–10 *μ*mol/kg) of MnDPDP. In a similar study Skjold et al. [17] assessed dose-responses in liver and left ventricular LV myocardium (Figure 8) with MnDPDP (5, 10, 15 *μ*mol/kg) administered outside magnet and intermittent recording of *R*_1_ over 24 hours.

Peak gains in *R*_1_ (Δ*R*_1_) above the native level were 35%, 40%, and 44% in LV myocardium whereas Δ*R*_1_ values were 3–6 times higher in liver. Myocardial *R*_1_ was stable for up to 3-4 hours, and still after 24 hours half of Δ*R*_1_ remained. In comparison, myocardial Δ*R*_1_ was considerably below that reported after injection (150 *μ*mol/kg) of gadopentetate dimeglumine (30%–74% at 2–20 min) [76] but moderately above that after infusion (5 *μ*mol/kg) of MnCl_2_ (23%) [78].

In LV myocardium, an optimal dose of MnDPDP (5–10 *μ*mol/kg) lowered *T*_1_ to midway (∼725 ms) between native values (1020 ms) and reported Gd-enhanced values (350–550 ms) [76]. Importantly, delayed MEMRI, highly feasible within 3-4 hours, provides an advantage for exploitation in patient turnover, in screening of viability, and potentially in theragnostic use of MnDPDP. In liver, a stable time window was shorter, 1-2 hours. The high tissue *R*_1_, however, makes it possible to quantify liver function and viability by a dose far lower than 5–10 *μ*mol/kg.

### 3.3. Analysis of Mn^2+^ Uptake

**Figure 9:**
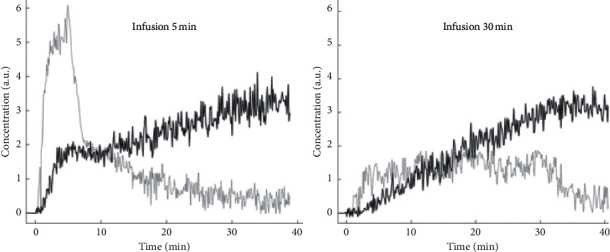
MnDPDP: myocardial Mn^2+^ uptake in healthy human volunteers [18]. MnDPDP 5 *μ*mol/kg was administered i.v. inside magnet with infusion time of 5 min (*n* = 5) or 30 min (*n* = 5). *R*_1_ values obtained at 1.5 T over 40 min after start of infusion were converted to tissue [Mn^2+^] in arbitrary units (a.u.). Δ*R*_1_ values were as follows: 5 min, 0.32 s^−1^; 30 min, 0.35 s^−1^ (reproduced with permission from J Magn Reson Imaging).

Myocardial Mn^2+^uptake from MnDPDP was monitored by continuous online recording of *R*_1_ in healthy young adults [18]. With the same dose (5 *μ*mol/kg), duration of infusion (Figure 9) presented different profiles for Δ*R*_1_ and Mn^2+^ uptake, biphasic (5 min) or linear (30 min). On the other hand, Δ*R*_1_ over 40 min did not differ between infusion groups (5 min, 0.32 s^−1^; 30 min, 0.35 s^−1^).

When a tracer kinetic model, based on cell influx of Mn^2+^ from an assumedly reversible (EC) into a largely irreversible (IC) compartment [79], was applied to the *R*_1_ curves, an unidirectional influx constant for Mn^2+^ (*K*_*i*_) was measured as an index of Ca^2+^ channel activity. As revealed in kinetic (Patlak) plots, the resulting *K*_*i*_ values (arbitrary units) were identical in the two infusion groups, 5 min (5.73) and 30 min (5.72). An attempt to measure tissue fraction of the Mn^2+^-donating compartment, i.e., the EC volume (ECV), revealed results far from an expected 25% level.

With adjustment of infusion time measurements of *K*_*i*_ and possibly of ECV, the latter a hallmark of Gd-based MRI [1, 80–82], may become exquisite tools in clinical physiology. It is also attractive to assess myocardial L-type Ca^2+^ channel activity [35], with contribution by other Ca^2+^ transporters [36, 37] in disease. Interestingly, the utility of MnDPDP in tracking Ca^2+^ channel activity has been confirmed in a meticulous study of retinal function in light- vs. dark-adapted rats [83].

### 3.4. Detection of Myocardial Ischemia by Stress Testing

In animals, MEMRI can detect myocardial ischemia on its own [9, 40] by revealing diminished Mn^2+^ uptake and Δ*R*_1_ in an ischemic region. Detection is strengthened, however, by infusion of the *β*-adrenergic agonist dobutamine which enhances inotropy and Mn^2+^ uptake in nonischemic remote regions. Efficacy of MEMRI in dobutamine testing requires highly mobile Mn^2+^ in plasma and interstitium, as was first demonstrated by Hu and Koretsky with MnCl_2_ in rats [12] and later confirmed by Eriksson and Johansson with a low affinity Mn-chelate in pigs [84]. With MnDPDP, however, Mn^2+^ release is too slow as documented by Amundsen et al. in human volunteers [71]. Hence, infusion of MnDPDP (5 *μ*mol/kg in 5 min) during dobutamine stress (10 min) did not raise myocardial *R*_1_ above the rest level.

Interestingly, native *T*_1_ mapping in patients with coronary artery disease [52] has shown that increases in myocardial blood volume (MBV) during vasodilation by adenosine, minimal in infarcted vs. maximal in remote regions, were paralleled by transient increases in *T*_1_ (0.2% *vs*. 6.2%). With infusion of adenosine in due time after MnDPDP infusion, an infarct-to-remote *T*_1_ gradient may be no less. Stress testing with adenosine after myocardial Mn^2+^ enhancement with MnDPDP may thus be an interesting option to pursue.

### 3.5. Cardiac Injury and Repair in Patients

Clinical reports with MnDPDP or other Mn^2+^-releasing agents concern cardiac remodeling following a previous AMI [19, 72–74]. In 2003, a congress abstract from Abolmaali et al. [72] reported that MnDPDP (10 *μ*mol/kg) reduced LV myocardial *T*_1_ at 1.5 T, from 550 ms to 450 ms in healthy volunteers (*n* = 9) and from 815 ms to 630 ms in patients with impending heart failure (*n* = 7). Unfortunately, these early data were not presented in a complete paper.

**Figure 10:**
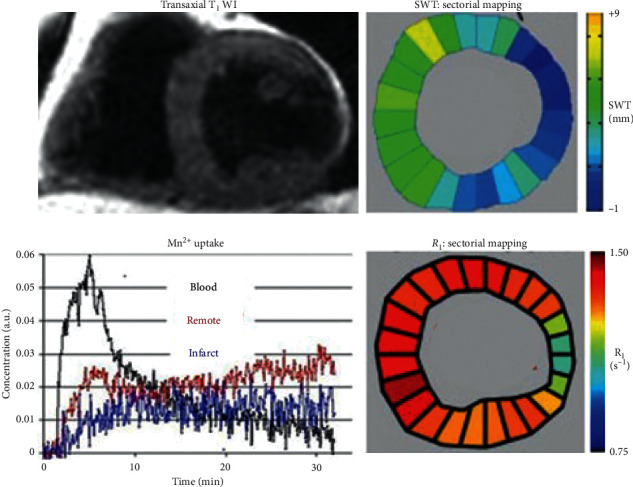
MnDPDP: myocardial remodeling in a patient examined 3 weeks after AMI treated with pPCI [19]. One hour after i.v. infusion of MnDPDP (5 *μ*mol/kg, 5 min) *T*_1_WI shows a transmural infarct in the LV lateral wall and an apparent thickening of remote myocardium. LV maps of SWT (mm) and of *R*_1_ (s^−1^) show parallel directions of rising values from the infarct towards remote sectors. Myocardial Mn^2+^ uptake (arbitrary units (a.u.)) over 30 min is biphasic in remote sectors and monophasic and smaller in the infarct. LV ejection fraction (LVEF): 48%. Reproduced with permission from J Magn Reson Imaging.

Present MRI techniques to describe the complex pathophysiology of cardiac remodeling [85–87] are based on signs of edema and fibrosis by delayed contrast enhancement with EC Gd agents or by native *T*_1_ mapping and detection of deficient contractile function by cine-MRI [1, 5, 80–82]. In 2007 Skjold et al. [19] applied MnDPDP to measure sector-wise myocardial viability by *R*_1_ and systolic wall thickening (SWT) in patients 3–12 weeks after AMI treated with primary Percutaneous Coronary Intervention (pPCI). Ten patients were examined by dual imaging, i.e., before and after i.v. infusion (5 min) of MnDPDP (5 *μ*mol/kg). *T*_1_WI after MnDPDP (Figure 10) demarcated infarcts in 4 patients only but revealed increase in remote wall thickening in 9. Importantly, in these 9 patients sectorial LV maps of *R*_1_ and SWT showed identical directions of growing infarct-to-remote gradients. Mn^2+^-uptake was biphasic in remote sectors but monophasic and smaller in the infarcted sectors. In one patient no change from normal appeared, and confirmed clinical indices of myocardial salvage.

**Figure 11:**
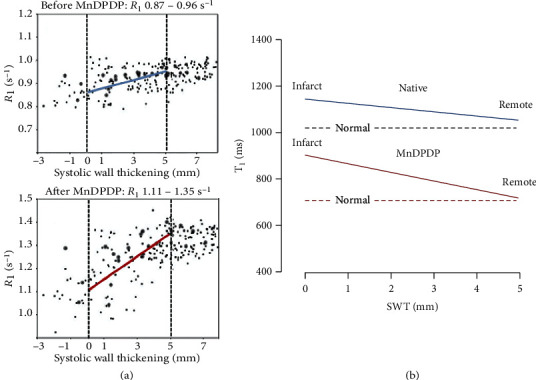
MnDPDP: myocardial remodeling—sectorial *R*_1_ (*T*_1_) vs. systolic wall thickening (SWT) [19]. Mapping of SWT and *R*_1_ at 1.5 T was undertaken in 24 sectors of LV myocardium before (native reference) and one hour after i.v. infusion of MnDPDP (5 *μ*mol/kg, 5 min). Data were obtained from 10 patients undergoing remodeling after AMI. (a) Measured values of *R*_1_ (s^−1^) vs. SWT (mm) before and after MnDPDP. Dotted black lines are drawn at SWT 0 and 5 mm; blue and red lines are drawn between mean *R*_1_ values at 0 and 5 mm SWT. In spite of large spread in individual *R*_1_ values, significant correlations were found between infarct-to-remote directional angles for SWT and *R*_1_ both before and after MnDPDP. Figure reproduced with permission from J Magn Reson Imaging. (b) Diagram based on values from (a) but presented as *T*_1_ (ms) vs. SWT (range 0–5 mm). The dotted horizontal lines mark *T*_1_ of normal myocardium [17, 18]. *T*_1_-SWT correlations are marked by continuous lines. Blue line: native *T*_1_ values (1150–1040 ms). Red line: *T*_1_ values after MnDPDP (900–740 ms).

A limitation to the above technique is the lack of finer details in *R*_1_ distribution since only a single mean *R*_1_ value represented each sector and more detailed *R*_1_ guided colour coding was not applied. Still, the accumulated data from all patients and sectors showed that SWT (range 0–5 mm) correlated significantly with both native *R*_1_ and *R*_1_ after MnDPDP. Moreover, infarct-to-remote *R*_1_ gradients (Figure 11(a)) were significant both before, 0.87–0.96 s^−1^ (Δ*R*_1_ 0.09 s^−1^), and after, 1.11–1.35 s^−1^ (Δ*R*_1_ 0.24 s^−1^), MnDPDP. These findings, as also presented in a *T*_1_–SWT diagram (Figure 11(b)), illustrate in a quantitative manner parallel but supplementary aspects of myocardial injury and remodeling. While native *T*_1_ maps present overall tissue conditions rather evenly [1, 81, 82] with main emphasis on EC events, *T*_1_ maps after Mn^2+^ enhancement encompass conditions in the major IC compartment. Accordingly, native MRI reflects edema plus fibrosis whereas MEMRI mainly reveals energy state and Ca^2+^ control in cardiomyocytes.

*R*_1_ elevation in revascularized infarct sectors with assumedly dead tissue (Figures 10 and 11) seemed a puzzling finding. Partial elevation of *R*_1_ in the infarct, as also observed in rat hearts (Figure 4), may, besides partial volume effects and Mn^2+^ uptake in scattered live cardiomyocytes, be caused by interstitial Mn^2+^ binding to connective tissue macromolecules. Another explanation is that Mn^2+^ may enter proliferating and Ca^2+^ conducting myofibroblasts which can uphold tensile strength and possess semicontractive properties in infarcted tissue [36, 37, 85–87]. Without delving into further mechanisms, mean sectorial *K*_*i*_ values for Mn^2+^ influx (arbitrary units) of 6.34 (remote) and 5.34 (infarct) and also mean sectorial ECV values of 25.8% (remote) and 35.1% (infarct) as reported by Skjold [74] may be consistent with active or hyperactive cardiomyocytes vs. tissue in extensive repair [85, 87].

Altogether, although small the study Skjold et al. provides a snapshot of how MEMRI might be exploited in the human heart. Both single imaging (MEMRI delayed or online) and dual imaging (native MRI + online MEMRI) may become attractive tools for an in-depth analysis of myocardial pathophysiology, not least when combined with more recently developed mapping techniques.

### 3.6. Experience with DEMRI plus MEMRI

In 2014, Matsuura et al. [73] reported dual contrast imaging in patients (*N* = 5) with ischemic cardiomyopathy using delayed enhancement MRI (DEMRI) with gadopentetate dimeglumine to be followed by MEMRI with use of EVP1001. The latter is a rapid Mn^2+^-releasing gluconate salt supplemented with Ca^2+^ (SeeMore™, Eagle Vision Pharmaceuticals, USA). The DEMRI, infarct plus peri-infarct (PIR), region and the infarcted MEMRI region measured by *T*_1_ mapping at 3.0 T revealed these volumes: DEMRI 34%, MEMRI 14%, and by subtraction PIR 20%. However, being effective in detecting the PIR for potential revascularization, the reported procedure required administering two contrast agents in two separate imaging sessions.

### 3.7. Recent Studies of MEMRI with MnDPDP in Animals

Two recent reports from *in vivo* rats deserve comment as they apply current techniques to provide up-to-date information on MnDPDP as a biomarker of widely differing tissue injuries.

In 2018, Spath et al. published an in vivo rat heart study [88] with measurement of myocardial infarct size (IS) 3 and 12 weeks after AMI. In introductory experiments, the *T*_1_ reducing capacity of EVP1001 (22 *μ*mol/kg) and MnCl_2_ (22 *μ*mol/kg) in normal myocardium at 7.0 T was twice that of MnDPDP (44 *μ*mol/kg). Still, AMI measurements of IS by use of EVP1001 (*n* = 6) and MnDPDP (*n* = 7) were obtained with equally high accuracy when compared to histology. DEMRI with gadobenate dimeglumine (500 *μ*mol/kg) applied in prior separate experiments was reported as less accurate than MEMRI in defining IS by including peri-infarct edema and fibrosis.

In 2020, Liu et al. [89] reported on the use of MnDPDP (25 *μ*mol/kg) and MEMRI to predict the therapeutic efficacy of a vascular disrupting anticancer agent (VDA) in rats with primary and secondary malignancies of liver. Tumor-to-liver contrast at 3.0 T was judged by tissue SI, and results were closely compared with postmortem microangiography and histology. VDA-mediated intratumoral necrosis was imaged by use of gadoterate meglumine (200 *μ*mol/kg).

**Figure 12:**
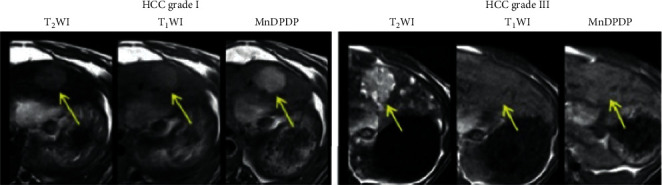
Predictive imaging prior to therapy of rat livers with hepatocellular carcinoma (HCC) of high (I) and low (III) grade of differentiation [89]. MnDPDP raised tumor-to-liver contrast in T_1_WIs, see arrow, in grade I HCC to the left, but hardly in grade III HCC as depicted to the right (reproduced with permission from Transl Oncol).

Important findings (Figure 12) were first that tumor-to-liver contrast enhancement by MnDPDP was strong in highly (grade I) and weak in lowly (grade III-IV) differentiated hepatocellular carcinoma (HCC) before treatment. Secondly, the necrotic responses to the VDA assessed by Gd-MRI correlated with the grade of differentiation, i.e., major in high and minor in low grade HCC. 24-hour delay in imaging after infusion of MnDPDP avoided transient blood pool effects and improved the contrast between the HCCs and liver. The study confirms that MEMRI with MnDPDP represents a noninvasive surrogate for biopsy taking in primary liver cancer.

## 4. Therapy in Humans

Three small scale feasibility studies [23−25] and one case report [22] indicate that MnDPDP may provide clinically relevant cytoprotection in humans.

### 4.1. AMI and Reperfusion Injury [25]

With the aim of preventing reperfusion injury during pPCI, patients submitted with their first episode of AMI were randomized to receive 2 min i.v. infusion of MnDPDP (2 *μ*mol/kg) or placebo (NaCl) immediately after angiography but prior to the reopening of a culprit coronary artery branch. The infusions were without side effects. As reported by Karlsson JE et al., the MnDPDP group revealed an unfavorable distribution of patients (Table 1), fewer intraventricular thrombi, and a trend towards more rapid reversal of ECG changes, but the remaining results did not reveal differences between groups. Thus, a tendency to potential benefit in few patients needs confirmation in a larger phase II trial, preferably based on an improved protocol.

### 4.2. Chemotherapy of Cancer and Adverse Events (AEs)

MnDPDP has been applied to patients with colorectal adenocarcinoma undergoing repeated treatment cycles with the platinum derivative oxaliplatin and 5-fluorouracil [22–24]. Severe adverse events (AEs) of oxaliplatin like painful acute or chronic peripheral sensory neuropathy (PSN) and bone marrow depression are closely related to oxidative stress [24, 62, 65, 66]. Importantly, chronic PSN may be caused by prooxidant platinum ions (Pt^2+^) accumulating in pain-conducting dorsal root ganglion cells [24].

#### 4.2.1. Case Report

**Figure 13:**
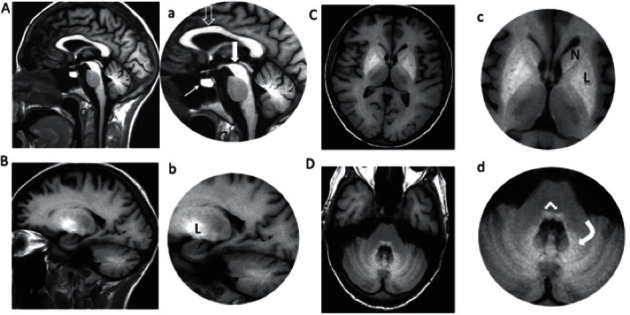
Brain MRI in a patient receiving MnDPDP 140 *μ*mol/kg over 8 months [22, 90]. MnDPDP (10 *μ*mol/kg) was applied as cytoprotective adjunct to 14 cycles of chemotherapy with oxaliplatin as the primary drug in a patient with cancer of colon. MRI of the brain (1.5 T) was undertaken after the last cycle. Sagittal and parasagittal images (A-B, a-b) were obtained by *T*_1_W-FLAIR and descending axial images (C-D, c-d) by *T*_1_W-SE. High SI reflects marked Mn deposition in: A-a, corpus callosum (open arrow), mesencephalon (thick white arrow), and pituitary gland (thin white arrow); B-b, C-c, putamen and globus pallidus (L nucleus lentiformis) and caput nucleus caudatus (N); D-d, cerebellum with nucleus dentatus (curved white arrow) and brain stem (white angled arrow) (Blomlie V, Jynge P., unpublished images).

The first patient to receive MnDPDP for therapy was a young male who received palliation by 14 cycles of oxaliplatin, each supplemented with MnDPDP 10 *μ*mol/kg, before he succumbed to disease [22]. The regimen went without PSN or reduction in white blood cell count (WBC), and there was a surprising lowering of pain. After 8 months, the patient developed a mild hand tremor as a potential early sign of Parkinsonism. Then, MRI of the brain (Figure 13) showed widely distributed Mn deposits [44, 45] with maximal SI in basal ganglia including dentate nucleus and globus pallidum. As recently discussed by Blomlie et al. [90] these basal ganglia sites are also noted for deposition of Gd^3+^ [91] indicating a common, possibly Ca^2+^ related, pathway for focal brain storage of these metals.

Mn deposition outside the basal ganglia indicated a most extensive brain overload due to additive predisposing factors: a too high total dose vs. time of MnDPDP; a marked influence by concomitant liver failure; and probably also a BBB weakened by disease and/or by chemotherapy [33, 44]. The case illustrates that, with a potential exception for end stage palliation, there is a need for dose reduction and attention to liver function and BBB integrity in multiple administrations of MnDPDP.

#### 4.2.2. Prevention of Acute Toxicity

**Figure 14:**
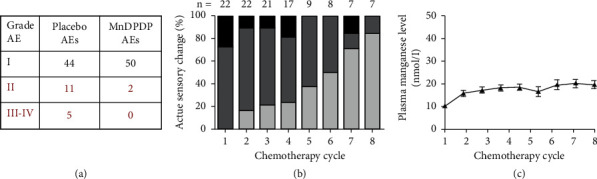
MnDPDP as cytoprotective adjunct to chemotherapy. Patients with advanced cancer of colon were treated with repeated cycles with oxaliplatin as primary anticancer drug and MnDPDP as adjunct for protection of normal tissues. (a) Adverse events (AEs) [23]. AEs of grade I (mild), II (moderate), III (severe), and IV (life-threatening) were recorded in 14 patients during 3 therapy cycles with oxaliplatin and with preinfusion of MnDPDP 2 *μ*mol/kg or saline (placebo). There was a major reduction in AEs grade II-IV with MnDPDP. Also plasma leukocyte content was maintained at a higher level with MnDPDP (reprinted with permission from *Translational Oncology*). (b) Peripheral sensory neuropathy (PSN) [24]. Patients that experienced PSN during previous oxaliplatin cycles were followed for up to 8 further cycles, each with preinfusion of MnDPDP 5 *μ*mol/kg. In these cycles, MnDPDP gradually reduced the initial severity of PSN (black > dark gray > light gray) indicating a reversal of the underlying nerve injuries (reprinted with permission from J Clin Invest). (c) Plasma [Mn] (nmol/L) during therapy with oxaliplatin and MnDPDP [24]. Patients cited in B showed a gradual rise in plasma [Mn] over 8 cycles in 4 months without exceeding normal levels of 10–20 nmol/L [29, 33] (reprinted with permission from J Clin Invest).

In the first feasibility study of cytoprotection of normal tissues, Karlsson et al. [23] examined a small group of patients with locally advanced cancer receiving 3 cycles of oxaliplatin, with each cycle preceded by a low dose of MnDPDP (2 *μ*mol/kg) or saline (placebo). Main significant findings with MnDPDP compared to placebo were a higher WBC after these cycles and almost absence of grade II-IV AEs. In particular, life threatening or severe AEs were only observed in the placebo group (Figure 14(a)).

#### 4.2.3. Prevention and Reversal of Neurotoxicity

In another feasibility study, Coriat et al. [24] examined patients with PSN already detected in prior oxaliplatin cycles who received 4–8 further cycles, but now with preinfusion of MnDPDP (5 *μ*mol/kg). After introducing MnDPDP, the PSNs became fewer and less severe (Figure 14(b)), indicating both prevention and reversal of nerve toxicity. These benefits were partly explained by acute MnSOD mimetic actions. Another likely mechanism implies chelation and elimination of oxidizing metals including platinum ions (Pt^2+^) released from oxaliplatin, an interpretation supported by EPR analysis revealing a Pt^2+^ affinity to DPDP close to that of Cu^+^ [92]. With an accumulated MnDPDP dose up to 40 *μ*mol/kg over 4 months in Coriat's study, plasma Mn (Figure 14(c)) rose gradually without exceeding normal levels [33]. There were no signs of Parkinsonism or bone marrow depression.

The two latter studies indicate that MnDPDP in a low imaging dose (2–5 *μ*mol/kg) at timely intervals (2–4 weeks) and with attention to liver function may prevent and reduce severe AEs in repeated (4–8) cycles of chemotherapy without causing any undue Mn accumulation as shown in the case report. The studies were too small, however, to indicate any effect upon tumor growth.

### 4.3. Experience with a Derivative of MnDPDP

[Ca_4_Mn(DPDP)_5_] (calmangafodipir, PledOx™, Aladote™, PledPharma AB, Sweden) was developed with the aim of combining efficacy in therapy with reduced brain Mn^2+^ uptake [59]. In a phase II trial, PledOx seemingly prevented oxaliplatin-induced PSN after 3 and 6 months of follow-up, but after 9 and 12 months, there were no differences between treated and nontreated groups [93]. In ongoing trials, paracetamol-overdose patients are given Aladote as supplement to the standard antidote N-acetyl-cysteine (NAC), and initial phase I data indicate suppression of early biomarkers of liver injury [94].

## 5. Back to the Future

In reappraising principle and agent for diagnostic imaging MEMRI and MnDPDP provide unique possibilities to quantify tissue function and viability at a cellular and subcellular level, with *T*_1_ mapping being more effective than *T*_1_WI. Administration of MnDPDP outside or inside the magnet enables examinations ranging from screening of heart disease and of arrhythmias to in-depth studies of cell Ca^2+^ fluxes and possibly measurement of ECV. Detailed information about injury, repair, and remodeling may also be obtained by dual imaging combining native MRI with MEMRI.

The above options may benefit from and potentially improve recent achievements in native MRI. With sharper delineation of cardiac anatomy, cine imaging and tagging of regional contractile function are distinct possibilities to exploit [51]. The same applies to myocardial *T*_1_ mapping in general and during adenosine stress to quantify MBV [52] or to measure perfusion by arterial spin labeling [95]. Hence, MEMRI with MnDPDP may give comprehensive information about myocardial viability, function, and perfusion, i.e., key indicators predicting the need for invasive coronary angiography or reducing the need for endomyocardial biopsies.

Against a future breakthrough speak a renewed position of Gd based MRI and the greater *T*_1_ shortening capacity of Gd agents compared to MnDPDP. In addition, recent improvements in native MRI may question the need for contrast agents [1, 51, 52, 82]. Notwithstanding, the IC approach with direct access to cardiomyocytes, multifunctional properties, and a potential to replace isotope scanning support a future role of cardiac MEMRI with MnDPDP. Likewise, quantification of viability is a unique principle which may be adopted for other organs like liver, pancreas, kidney, endocrine, and exocrine glands, subjected to tissue injury and repair.

Of particular advantage is that cytoprotection offered by MnDPDP may both increase the safety and extend the diagnostic applications. A major problem in cardiovascular disease and in diabetes refers to the use of contrast media in patients with impaired kidney function. At present, the intravascular, nanoparticular, and iron oxide-containing compound Ferumoxytol, mainly a *T*_2_ or *T*_2_^*∗*^ agent, serves as a safe substitute for Gd compounds in MRI of kidney [96]. Interestingly, with transient renal perfusion with MnDPDP including MnPLED and uptake/retention of paramagnetic Mn^2+^ in the cortex, MnDPDP might become attractive as a safe alternative. What is essential for safety is conservation of NO, a mediator of intrarenal perfusion and key to kidney preservation [97]. With an apparent cortex-to-medulla *T*_1_ gradient and long imaging window [13, 16], MnDPDP might also be effective in imaging of renal diseases. Altogether, combining imaging with potential tissue protection, hitherto not tested in the human kidney, may become an important option to pursue.

Since MnDPDP both images and preserves viable myocardium, theragnostic use seems a distinct possibility, for example, in AMI, the post-cardiac-arrest syndrome, and heart failure with inflammation and oxidative stress. A particularly important scenario may be its use as cytoprotective and diagnostic adjunct to chemotherapy with anthracyclines [58, 70, 98] which cause both acute and chronic heart failure at least partly due to production of ROS-RNS. In spite of limited or no success with scavenging agents [98], it still seems rational to attack the problem with a potent catalytic antioxidant acting at both initial and subsequent steps in a prooxidant cascade. MnDPDP may here be given as a cytoprotectant at onset of each treatment cycle while serving as a contrast agent for delayed imaging and monitoring of myocardial viability.

A parallel indication concerns the liver in abdominal cancer. In hepatic failure induced by paracetamol [66, 94] or by other etiology (hepatitis), low-dose MnDPDP may become both therapeutic drug and biomarker. A further option is in the transplantation field with imaging and protection of donor cells and organs as well as of the recipient. Stem cells in general [99] and pancreatic islets [100] together with cardiac, liver, and kidney transplants might become likely candidates.

“Manganese and MRI” reveals a current annual publication rate of about 100, but with more focus on new and stable macrocyclic chelates or (nano)particulate matter than on Mn^2+^-releasing agents as is required in MEMRI. Thus Mn^2+^ apparently substitutes for Gd^3+^ in novel highly stable complexes designed for EC, intravascular, or molecular-targeted deliveries [101, 102]. With exception of EVP1001 [73] MEMRI has not materialized in new *i.v.* formulations for trial in humans. Of considerable interest, though, is the recent indication in animals [103] of efficacy of a miniature dose of a ^52^Mn tracer with MEMRI-like properties in PET of the brain, thereby offering promise for functional PET/MRI.

## 6. Conclusion

Attempts are now made to reposition MnDPDP for diagnostic use in both the USA [46] and Europe [104]. With current insight into its work mode in MEMRI and in treating conditions of oxidative stress, previous indications are open for immediate use and new possibilities appear ready for off-label assessment of a future potential. The challenge will be to develop MEMRI and MnDPDP for use in daily routine and not only as exciting tools in clinical research. Thorough clinical trials are thus required.

## Figures and Tables

**Table 1:** Therapy with MnDPDP: cardioprotective adjunct to pPCI during AMI [25].

Group	Ischemia time (min)	TIMI flow grade I before reflow (patients)	STER (%)	CK-MB (*μ*g/L)	LVEF (%)	Infarct size (%)	LV thrombi (patients)
Placebo (*n* = 10)	144	3 of 10	73.1	4850	41.8	32.5	5 of 8
MnDPDP (*n* = 10)	206	0 of 10	84.3	4730	47.7	26.2	1 of 10
*p* value	0.04	0.07	0.08	0.75	0.50	0.62	0.02

Data are expressed as mean with *p* values (two-tailed) included. Data in three rows to the right were obtained by the use of late Gd-enhancement MRI (gadopentetate dimeglumine). TIMI, grading of coronary flow from 0 to 3; STER, ST segment elevation regression at 48 hours; CK-MB, plasma creatine kinase isoenzyme MB 0–48 hours; LVEF: LV ejection fraction.
